# 

**Published:** 1895-04-06

**Authors:** 


					HOSPITAL. J April 6th, 1895.
WITH EXTRA NURSING SUPPLEMENT.
Per Annum, 10s. 8d.. post free.
Monthly Parts, Od.; post free, Bd.
Ditto per Annum, 8s. 6d., post free.
Lyiscoi* Western Infirm am -t-~~ ^~ '?'**' \JoMfou<Vuo ncrhich hospi t*l
No. 445. Vol. XVIII. WITH EXTRA NUR8IN8 SUPPLEMENT. Sattbday, April 6th, 1895.

				

